# 1,1,1-Trichloro-2,2-bis­(4-eth­oxy­phen­yl)ethane

**DOI:** 10.1107/S1600536812043826

**Published:** 2012-10-27

**Authors:** Graham Smith

**Affiliations:** a Science and Engineering Faculty, Queensland University of Technology, GPO Box 2434, Brisbane, Queensland 4001, Australia

## Abstract

In the title compound, C_18_H_19_Cl_3_O_2_, which is the 4-eth­oxy­phenyl analogue of the insecticidally active 4-meth­oxy­phenyl compound meth­oxy­chlor, the dihedral angle between the two benzene rings is 60.38 (13)°. An intra­molecular aromatic C—H⋯Cl inter­action is present.

## Related literature

For background to DDT-type insecticides, see: Läuger *et al.* (1944 ▶). For unit-cell data for the title compound, see: Schneider & Fankuchen (1946 ▶). For the structures of analogous *p*-alk­oxy-substituted DDT compounds, see: Smith *et al.* (1976 ▶); Smith (2012 ▶).
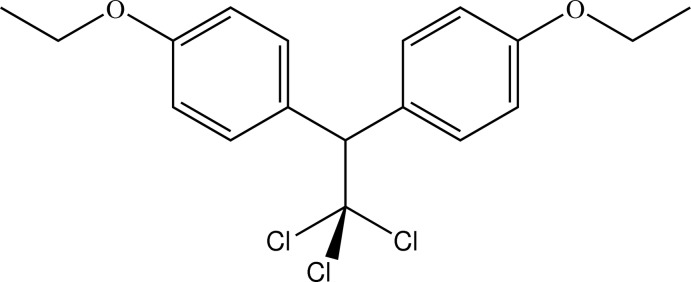


## Experimental

### 

#### Crystal data

C_18_H_19_Cl_3_O_2_
*M* *_r_* = 373.68
Monoclinic, 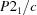

*a* = 23.4405 (7) Å
*b* = 9.8835 (2) Å
*c* = 7.7924 (2) Å
β = 99.536 (3)°
*V* = 1780.35 (8) Å^3^
*Z* = 4
Mo *K*α radiation
μ = 0.52 mm^−1^
*T* = 200 K
0.30 × 0.15 × 0.08 mm

#### Data collection

Oxford Diffraction Gemini-S CCD-detector diffractometer
Absorption correction: multi-scan (*CrysAlis PRO*; Agilent, 2012 ▶) *T*_min_ = 0.960, *T*_max_ = 0.980
10942 measured reflections
3109 independent reflections
2282 reflections with *I* > 2σ(*I*)
*R*_int_ = 0.106

#### Refinement

*R*[*F* ^2^ > 2σ(*F* ^2^)] = 0.046
*wR*(*F* ^2^) = 0.092
*S* = 0.91
3109 reflections
210 parameters
H-atom parameters constrained
Δρ_max_ = 0.38 e Å^−3^
Δρ_min_ = −0.25 e Å^−3^

### 

Data collection: *CrysAlis PRO* (Agilent, 2012 ▶); cell refinement: *CrysAlis PRO*; data reduction: *CrysAlis PRO*; program(s) used to solve structure: *SIR92* (Altomare *et al.*, 1993 ▶); program(s) used to refine structure: *SHELXL97* (Sheldrick, 2008 ▶) within *WinGX* (Farrugia, 1999 ▶); molecular graphics: *PLATON* (Spek, 2009 ▶); software used to prepare material for publication: *PLATON*.

## Supplementary Material

Crystal structure: contains datablock(s) global, I. DOI: 10.1107/S1600536812043826/lh5546sup1.cif

Structure factors: contains datablock(s) I. DOI: 10.1107/S1600536812043826/lh5546Isup2.hkl

Supplementary material file. DOI: 10.1107/S1600536812043826/lh5546Isup3.cml

Additional supplementary materials:  crystallographic information; 3D view; checkCIF report

## Figures and Tables

**Table 1:** Hydrogen-bond geometry (Å, °)

*D*—H⋯*A*	*D*—H	H⋯*A*	*D*⋯*A*	*D*—H⋯*A*
C2*B*—H2*B*⋯Cl2	0.93	2.67	3.321 (3)	128

**Fig. 1.**
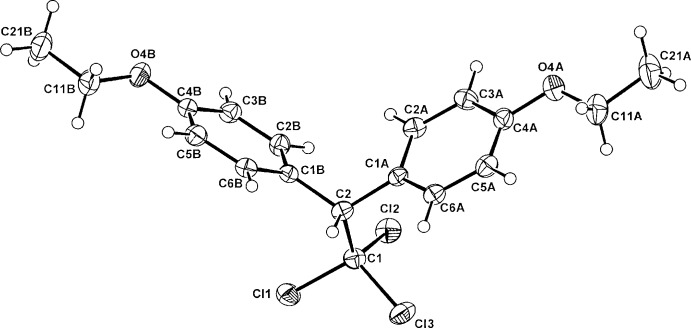
The molecular structure of the title compound, with displacement ellipsoids drawn at the 50% probability level.

**Fig. 2.**
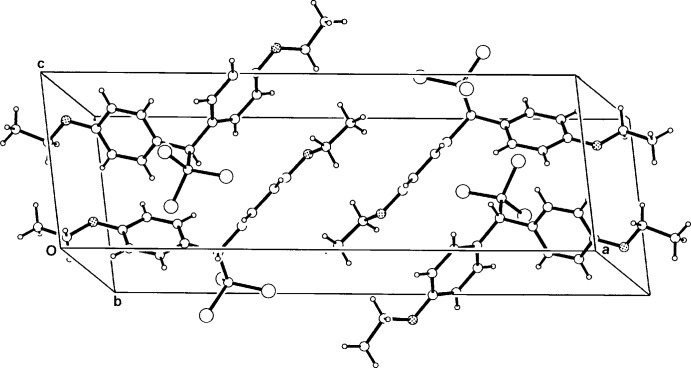
A perspective view of the crystal packing in the unit cell viewed approximately along the b axis.

## supplementary crystallographic information

### Comment

The title compound, C_18_H_19_Cl_3_0_2_ is the 4-ethoxyphenyl analogue of the insecticides DDT [1,1,1-trichloro-2,2-bis(4-chlorophenyl)ethane] and methoxychlor (the 4-methoxyphenyl analogue), and has similar insecticidal activity (Läuger *et al.*, 1944), but was not used as a commercial product. The crystal structures of methoxychlor (Smith *et al.*, 1976) and the *p*-butoxy analogue (Smith, 2012) are known and the unit cell data for the title compound has been reported (Schneider & Fankuchen, 1946). The structure of the title compound is reported herein.

The molecular structure of the title compound is shown in Fig. 1. The unit cell and space group are consistent with those previously reported. The dihedral angle between the two benzene ring mean planes is 60.38 (13)°. The value of this angle is 77.7° (no s.u. available) in the structure of methoxychlor (Smith *et al.*, 1976). The conformations of the two ethoxy side chains relative to their benzene rings (*A* and *B*) are similar [comparative torsion angles C3—C4—O4—C11, -173.3 (3) and 162.2 (2), respectively]. The *B* ring conformation is stabilized by an intramolecular aromatic C2*B*—H···Cl2 interaction (Table 1). No significant intermolecular interactions are present (Fig. 2).

### Experimental

The title compound was obtained as an analytical reference standard from the US Public Health Service. Colourless crystal prisms suitable for X-ray analysis were obtained by room temperature evaporation of a solution of the title compound in ethanol.

### Refinement

Hydrogen atoms were included in the refinement at calculated positions [C—H = 0.93–0.98 Å, with *U*_iso_(H) = 1.2*U*_eq_(C)(aromatic, methylene and methine) or 1.5*U*_eq_(*C*)(methyl), using a riding-model approximation.

### Crystal data

**Table:**

C_18_H_19_Cl_3_O_2_	*F*(000) = 776
*M**_r_* = 373.68	*D*_x_ = 1.394 Mg m^−^^3^
Monoclinic, *P*2_1_/*c*	Mo *K*α radiation, λ = 0.71073 Å
Hall symbol: -P 2ybc	Cell parameters from 3070 reflections
*a* = 23.4405 (7) Å	θ = 3.1–28.8°
*b* = 9.8835 (2) Å	µ = 0.52 mm^−^^1^
*c* = 7.7924 (2) Å	*T* = 200 K
β = 99.536 (3)°	Prism, colourless
*V* = 1780.35 (8) Å^3^	0.30 × 0.15 × 0.08 mm
*Z* = 4	

### Data collection

**Table:**

Oxford Diffraction Gemini-S CCD-detector diffractometer	3109 independent reflections
Radiation source: Enhance (Mo) X-ray source	2282 reflections with *I* > 2σ(*I*)
Graphite monochromator	*R*_int_ = 0.106
Detector resolution: 16.077 pixels mm^-1^	θ_max_ = 25.0°, θ_min_ = 3.4°
ω scans	*h* = −27→23
Absorption correction: multi-scan (*CrysAlis PRO*; Agilent, 2012)	*k* = −11→11
*T*_min_ = 0.960, *T*_max_ = 0.980	*l* = −9→9
10942 measured reflections	

### Refinement

**Table:**

Refinement on *F*^2^	Primary atom site location: structure-invariant direct methods
Least-squares matrix: full	Secondary atom site location: difference Fourier map
*R*[*F*^2^ > 2σ(*F*^2^)] = 0.046	Hydrogen site location: inferred from neighbouring sites
*wR*(*F*^2^) = 0.092	H-atom parameters constrained
*S* = 0.91	*w* = 1/[σ^2^(*F*_o_^2^) + (0.013*P*)^2^] where *P* = (*F*_o_^2^ + 2*F*_c_^2^)/3
3109 reflections	(Δ/σ)_max_ < 0.001
210 parameters	Δρ_max_ = 0.38 e Å^−^^3^
0 restraints	Δρ_min_ = −0.25 e Å^−^^3^

### Special details

**Table:**

Geometry. Bond distances, angles *etc*. have been calculated using the rounded fractional coordinates. All su's are estimated from the variances of the (full) variance-covariance matrix. The cell e.s.d.'s are taken into account in the estimation of distances, angles and torsion angles
Refinement. Refinement of *F*^2^ against ALL reflections. The weighted *R*-factor *wR* and goodness of fit *S* are based on *F*^2^, conventional *R*-factors *R* are based on *F*, with *F* set to zero for negative *F*^2^. The threshold expression of *F*^2^ > σ(*F*^2^) is used only for calculating *R*-factors(gt) *etc*. and is not relevant to the choice of reflections for refinement. *R*-factors based on *F*^2^ are statistically about twice as large as those based on *F*, and *R*- factors based on ALL data will be even larger.

### Fractional atomic coordinates and isotropic or equivalent isotropic displacement parameters (Å^2^)

**Table:**

	*x*	*y*	*z*	*U*_iso_*/*U*_eq_	
Cl1	0.33099 (3)	−0.05016 (8)	0.40168 (9)	0.0385 (3)	
Cl2	0.23442 (3)	−0.19217 (7)	0.50376 (9)	0.0374 (3)	
Cl3	0.21520 (3)	0.01715 (8)	0.24488 (9)	0.0412 (3)	
O4A	0.02901 (9)	0.0931 (2)	0.7508 (3)	0.0442 (8)	
O4B	0.44289 (8)	0.0498 (2)	1.1563 (2)	0.0406 (7)	
C1	0.25902 (12)	−0.0312 (3)	0.4447 (3)	0.0274 (9)	
C1A	0.19612 (11)	0.0869 (3)	0.6338 (3)	0.0228 (9)	
C1B	0.30558 (11)	0.0680 (3)	0.7397 (3)	0.0247 (9)	
C2	0.25640 (11)	0.0785 (3)	0.5851 (3)	0.0240 (9)	
C2A	0.17938 (12)	0.0081 (3)	0.7642 (3)	0.0293 (10)	
C2B	0.31987 (12)	−0.0486 (3)	0.8366 (3)	0.0293 (9)	
C3A	0.12369 (13)	0.0129 (3)	0.7994 (4)	0.0325 (10)	
C3B	0.36559 (12)	−0.0512 (3)	0.9749 (4)	0.0322 (10)	
C4A	0.08312 (12)	0.0979 (3)	0.7062 (4)	0.0304 (10)	
C4B	0.39827 (12)	0.0635 (3)	1.0180 (3)	0.0301 (10)	
C5A	0.09942 (12)	0.1816 (3)	0.5803 (3)	0.0316 (10)	
C5B	0.38476 (12)	0.1820 (3)	0.9249 (3)	0.0314 (10)	
C6A	0.15553 (12)	0.1748 (3)	0.5458 (3)	0.0291 (9)	
C6B	0.33864 (12)	0.1830 (3)	0.7873 (3)	0.0273 (9)	
C11A	−0.01660 (13)	0.1672 (3)	0.6474 (4)	0.0485 (11)	
C11B	0.48754 (13)	0.1495 (3)	1.1746 (4)	0.0425 (11)	
C21A	−0.07174 (14)	0.1329 (4)	0.7104 (5)	0.0641 (16)	
C21B	0.53406 (13)	0.1062 (3)	1.3219 (4)	0.0509 (14)	
H2	0.26230	0.16490	0.52880	0.0290*	
H2A	0.20620	−0.04910	0.82900	0.0350*	
H2B	0.29830	−0.12680	0.80820	0.0350*	
H3A	0.11330	−0.04150	0.88660	0.0390*	
H3B	0.37420	−0.13040	1.03840	0.0390*	
H5A	0.07300	0.24180	0.51950	0.0380*	
H5B	0.40630	0.26010	0.95420	0.0380*	
H6A	0.16620	0.23110	0.46090	0.0350*	
H6B	0.32970	0.26270	0.72540	0.0330*	
H11A	−0.01930	0.14280	0.52580	0.0580*	
H11B	0.47210	0.23700	1.20010	0.0510*	
H12A	−0.00910	0.26360	0.65890	0.0580*	
H12B	0.50340	0.15690	1.06760	0.0510*	
H21A	−0.10340	0.17870	0.63990	0.0960*	
H21B	0.56480	0.17140	1.33640	0.0760*	
H22A	−0.06920	0.16110	0.82930	0.0960*	
H22B	0.54890	0.01940	1.29560	0.0760*	
H23A	−0.07800	0.03700	0.70230	0.0960*	
H23B	0.51810	0.10010	1.42730	0.0760*	

### Atomic displacement parameters (Å^2^)

**Table:**

	*U*^11^	*U*^22^	*U*^33^	*U*^12^	*U*^13^	*U*^23^
Cl1	0.0348 (5)	0.0443 (5)	0.0374 (5)	0.0061 (4)	0.0093 (3)	−0.0034 (4)
Cl2	0.0452 (5)	0.0235 (4)	0.0428 (5)	−0.0032 (3)	0.0053 (4)	−0.0059 (3)
Cl3	0.0453 (5)	0.0481 (5)	0.0263 (4)	0.0079 (4)	−0.0054 (3)	−0.0015 (4)
O4A	0.0280 (13)	0.0556 (15)	0.0488 (14)	0.0015 (11)	0.0054 (10)	−0.0016 (11)
O4B	0.0327 (12)	0.0469 (14)	0.0373 (12)	−0.0064 (10)	−0.0081 (10)	0.0016 (10)
C1	0.0303 (17)	0.0255 (16)	0.0252 (15)	0.0056 (13)	0.0014 (13)	0.0001 (13)
C1A	0.0249 (16)	0.0193 (14)	0.0230 (15)	0.0016 (12)	0.0005 (12)	−0.0031 (12)
C1B	0.0250 (16)	0.0248 (15)	0.0244 (15)	0.0002 (13)	0.0044 (12)	0.0024 (13)
C2	0.0288 (16)	0.0162 (14)	0.0254 (15)	0.0014 (12)	−0.0003 (12)	0.0003 (12)
C2A	0.0333 (18)	0.0210 (15)	0.0316 (17)	0.0031 (13)	−0.0001 (14)	0.0034 (13)
C2B	0.0276 (17)	0.0269 (15)	0.0314 (17)	−0.0041 (13)	−0.0007 (13)	0.0021 (13)
C3A	0.0357 (18)	0.0270 (16)	0.0350 (18)	−0.0015 (14)	0.0062 (14)	0.0031 (14)
C3B	0.0337 (18)	0.0307 (17)	0.0315 (17)	0.0014 (14)	0.0030 (14)	0.0064 (14)
C4A	0.0262 (17)	0.0338 (17)	0.0303 (17)	−0.0023 (14)	0.0025 (14)	−0.0093 (14)
C4B	0.0289 (17)	0.0385 (18)	0.0219 (16)	0.0016 (14)	0.0014 (13)	−0.0041 (14)
C5A	0.0319 (18)	0.0286 (17)	0.0312 (17)	0.0052 (14)	−0.0036 (14)	0.0021 (14)
C5B	0.0306 (17)	0.0301 (17)	0.0328 (17)	−0.0063 (14)	0.0036 (14)	−0.0070 (14)
C6A	0.0347 (18)	0.0250 (15)	0.0262 (16)	−0.0008 (13)	0.0007 (13)	0.0023 (13)
C6B	0.0302 (17)	0.0251 (16)	0.0268 (16)	0.0010 (13)	0.0051 (13)	0.0018 (13)
C11A	0.0314 (19)	0.050 (2)	0.061 (2)	0.0063 (17)	−0.0013 (17)	−0.0054 (18)
C11B	0.0329 (19)	0.051 (2)	0.0425 (19)	−0.0057 (16)	0.0028 (15)	−0.0063 (16)
C21A	0.029 (2)	0.065 (3)	0.097 (3)	−0.0024 (19)	0.007 (2)	−0.013 (2)
C21B	0.033 (2)	0.066 (3)	0.048 (2)	−0.0009 (17)	−0.0103 (16)	−0.0111 (18)

### Geometric parameters (Å, º)

**Table:**

Cl1—C1	1.784 (3)	C11A—C21A	1.496 (5)
Cl2—C1	1.779 (3)	C11B—C21B	1.508 (4)
Cl3—C1	1.783 (3)	C2—H2	0.9800
O4A—C4A	1.370 (4)	C2A—H2A	0.9300
O4A—C11A	1.429 (4)	C2B—H2B	0.9300
O4B—C4B	1.379 (3)	C3A—H3A	0.9300
O4B—C11B	1.427 (4)	C3B—H3B	0.9300
C1—C2	1.549 (4)	C5A—H5A	0.9300
C1A—C2	1.525 (4)	C5B—H5B	0.9300
C1A—C2A	1.388 (4)	C6A—H6A	0.9300
C1A—C6A	1.384 (4)	C6B—H6B	0.9300
C1B—C2	1.527 (3)	C11A—H11A	0.9700
C1B—C2B	1.388 (4)	C11A—H12A	0.9700
C1B—C6B	1.391 (4)	C11B—H11B	0.9700
C2A—C3A	1.379 (4)	C11B—H12B	0.9700
C2B—C3B	1.389 (4)	C21A—H21A	0.9600
C3A—C4A	1.382 (4)	C21A—H22A	0.9600
C3B—C4B	1.378 (4)	C21A—H23A	0.9600
C4A—C5A	1.385 (4)	C21B—H21B	0.9600
C4B—C5B	1.386 (4)	C21B—H22B	0.9600
C5A—C6A	1.387 (4)	C21B—H23B	0.9600
C5B—C6B	1.391 (4)		
			
C4A—O4A—C11A	118.5 (2)	C3A—C2A—H2A	119.00
C4B—O4B—C11B	117.3 (2)	C1B—C2B—H2B	119.00
Cl1—C1—Cl2	108.18 (16)	C3B—C2B—H2B	119.00
Cl1—C1—Cl3	106.86 (13)	C2A—C3A—H3A	120.00
Cl1—C1—C2	110.84 (19)	C4A—C3A—H3A	120.00
Cl2—C1—Cl3	107.53 (15)	C2B—C3B—H3B	120.00
Cl2—C1—C2	113.01 (18)	C4B—C3B—H3B	120.00
Cl3—C1—C2	110.17 (19)	C4A—C5A—H5A	120.00
C2—C1A—C2A	122.5 (2)	C6A—C5A—H5A	120.00
C2—C1A—C6A	120.0 (2)	C4B—C5B—H5B	120.00
C2A—C1A—C6A	117.4 (2)	C6B—C5B—H5B	120.00
C2—C1B—C2B	124.6 (3)	C1A—C6A—H6A	119.00
C2—C1B—C6B	118.0 (2)	C5A—C6A—H6A	119.00
C2B—C1B—C6B	117.4 (2)	C1B—C6B—H6B	119.00
C1—C2—C1A	111.2 (2)	C5B—C6B—H6B	119.00
C1—C2—C1B	113.4 (2)	O4A—C11A—H11A	110.00
C1A—C2—C1B	114.7 (2)	O4A—C11A—H12A	110.00
C1A—C2A—C3A	121.2 (3)	C21A—C11A—H11A	110.00
C1B—C2B—C3B	121.6 (3)	C21A—C11A—H12A	110.00
C2A—C3A—C4A	120.6 (3)	H11A—C11A—H12A	108.00
C2B—C3B—C4B	120.0 (3)	O4B—C11B—H11B	110.00
O4A—C4A—C3A	115.5 (3)	O4B—C11B—H12B	110.00
O4A—C4A—C5A	125.2 (3)	C21B—C11B—H11B	110.00
C3A—C4A—C5A	119.3 (3)	C21B—C11B—H12B	110.00
O4B—C4B—C3B	115.4 (2)	H11B—C11B—H12B	109.00
O4B—C4B—C5B	124.8 (3)	C11A—C21A—H21A	109.00
C3B—C4B—C5B	119.9 (2)	C11A—C21A—H22A	109.00
C4A—C5A—C6A	119.4 (3)	C11A—C21A—H23A	109.00
C4B—C5B—C6B	119.4 (3)	H21A—C21A—H22A	109.00
C1A—C6A—C5A	122.1 (2)	H21A—C21A—H23A	109.00
C1B—C6B—C5B	121.8 (3)	H22A—C21A—H23A	110.00
O4A—C11A—C21A	107.8 (3)	C11B—C21B—H21B	109.00
O4B—C11B—C21B	107.8 (2)	C11B—C21B—H22B	109.00
C1—C2—H2	106.00	C11B—C21B—H23B	110.00
C1A—C2—H2	106.00	H21B—C21B—H22B	109.00
C1B—C2—H2	106.00	H21B—C21B—H23B	109.00
C1A—C2A—H2A	119.00	H22B—C21B—H23B	109.00
			
C11A—O4A—C4A—C3A	−173.3 (3)	C2B—C1B—C2—C1	−52.8 (3)
C11A—O4A—C4A—C5A	8.0 (4)	C2B—C1B—C2—C1A	76.3 (3)
C4A—O4A—C11A—C21A	173.7 (3)	C6B—C1B—C2—C1	127.1 (3)
C11B—O4B—C4B—C3B	162.2 (2)	C6B—C1B—C2—C1A	−103.8 (3)
C11B—O4B—C4B—C5B	−18.4 (4)	C2—C1B—C2B—C3B	179.4 (3)
C4B—O4B—C11B—C21B	−174.3 (2)	C6B—C1B—C2B—C3B	−0.5 (4)
Cl1—C1—C2—C1A	−175.16 (18)	C2—C1B—C6B—C5B	−179.1 (2)
Cl1—C1—C2—C1B	−44.3 (3)	C2B—C1B—C6B—C5B	0.8 (4)
Cl2—C1—C2—C1A	−53.5 (3)	C1A—C2A—C3A—C4A	−0.7 (4)
Cl2—C1—C2—C1B	77.3 (3)	C1B—C2B—C3B—C4B	−0.5 (4)
Cl3—C1—C2—C1A	66.8 (2)	C2A—C3A—C4A—O4A	179.2 (3)
Cl3—C1—C2—C1B	−162.36 (19)	C2A—C3A—C4A—C5A	−2.0 (4)
C2A—C1A—C2—C1	88.5 (3)	C2B—C3B—C4B—O4B	−179.5 (2)
C2A—C1A—C2—C1B	−41.7 (4)	C2B—C3B—C4B—C5B	1.1 (4)
C6A—C1A—C2—C1	−91.0 (3)	O4A—C4A—C5A—C6A	−179.0 (3)
C6A—C1A—C2—C1B	138.9 (3)	C3A—C4A—C5A—C6A	2.4 (4)
C2—C1A—C2A—C3A	−176.7 (3)	O4B—C4B—C5B—C6B	179.9 (2)
C6A—C1A—C2A—C3A	2.8 (4)	C3B—C4B—C5B—C6B	−0.8 (4)
C2—C1A—C6A—C5A	177.1 (2)	C4A—C5A—C6A—C1A	−0.2 (4)
C2A—C1A—C6A—C5A	−2.4 (4)	C4B—C5B—C6B—C1B	−0.2 (4)

### Hydrogen-bond geometry (Å, º)

**Table:**

*D*—H···*A*	*D*—H	H···*A*	*D*···*A*	*D*—H···*A*
C2*B*—H2*B*···Cl2	0.93	2.67	3.321 (3)	128
